# Health Care Workers' Knowledge, Attitudes, and Beliefs Related to COVID-19 in Palliative Medicine and Hospice Care

**DOI:** 10.1089/pmr.2020.0115

**Published:** 2020-12-23

**Authors:** Zaida Albarracin, Michael Silverman, Jocelyn Mineo, Baher Al-Abbasi, Susan Koff, Catherine Martell, Richard S. Levene

**Affiliations:** ^1^ University of Miami/John F. Kennedy (JFK) Medical Center, Atlantis, Florida, USA.; ^2^ Geriatrics/Palliative Medicine/Hospice, West Palm Beach VA Medical Center, West Palm Beach, Florida, USA.; ^3^ HCA Healthcare: East Florida Division GME, Ft. Lauderdale, Florida, USA.; ^4^ HCA Physician Services Group, East Florida Division, Ft. Lauderdale, Florida, USA.; ^5^ Trustbridge Health, West Palm Beach, Florida, USA.

**Keywords:** attitudes, beliefs, COVID-19, hospice, knowledge, palliative

## Abstract

***Background:*** Although coronavirus disease 2019 (COVID-19) has impacted on a global scale, the knowledge, attitudes, and beliefs of the health care workers who provide the care at the end of life have not been evaluated.

***Objectives:*** To assess and understand palliative medicine and hospice care health care workers' knowledge, attitudes, and beliefs related to COVID-19.

***Design:*** A web-based survey was created. Primary outcomes included attitudes, beliefs, and knowledge. Secondary outcomes included comparison in between health care workers who described themselves at high risk versus not at high risk of complications related to COVID-19 infection.

***Setting/Subjects:*** In total, 1262 adult hospice workers in the United States were invited.

***Results:*** A total of 348 workers completed the survey. Of them, 321 were analyzed, 54.52% were over the age of 50 years, 84.74% were females, 41.75% were nurses, 29.6% were administrative staff, and 6.54% were physicians. Of these workers, 39.56% considered themselves at high risk to develop complications related to COVID-19 infection, 74.46% felt neutral to uncomfortable treating these patients, 77.57% believed that the recommended personal protective equipment (PPE) was adequate, 89.41% supported the risk-reduction strategies, 84.73% obtained information from health authorities, 25.55% from social media, 31.46% believed COVID-19 was likely created in a laboratory or intentionally, and 66.14% of hospice workers who considered themselves at high risk of complications felt available PPE was adequate to protect them compared with 85.05% of responders who did not consider themselves at high risk (*p* < 0.0001). The majority of respondents were incorrect in seven of the eight clinical scenarios.

***Conclusion:*** Improving staff knowledge and information related to COVID-19 would enhance staff safety, improve patient care, and relieve anxiety.

## Introduction

The coronavirus disease 2019 (COVID-19) pandemic has had a global impact and highlighted the vulnerability of an aging population to highly contagious diseases.^1,2^ The pandemic has spread devastation to millions of people in the United States and has created a major challenge to the health care system.^3^ The elderly have suffered significantly, with a disproportionate number of deaths.^4–6^ Deaths among health care workers infected with COVID-19 are rare and have mostly affected those greater than age 50 years with underlying medical conditions.^7,8^ Palliative medicine and hospice personnel provide a major role in the care of COVID-19 patients. In the first days to weeks of hospitalization, these personnel are helping with complex advance care planning conversations, while also adding an expert layer of support for symptom management.^9,10^ They also help with communication and provide emotional support to the patients and their families who cannot be present, in particular, those with progressive disease whom are designated as terminal.^11^ Thus, it is imperative to better understand the knowledge, attitudes, and beliefs of these health care providers regarding COVID-19 as it could negatively impact patient care at the end of life.

Accurate and up-to-date information for health care workers is of critical importance during the COVID-19 pandemic. Unfortunately, in modern society, there are many conflicting sources of information, much of it being misinformation, creating confusion and fear. Within the United States governmental structure, the numerous levels of governing bodies and regulatory agencies can often produce conflicting guidelines. Public health recommendations by international sources may be as important to understand as a local recommendation given the nature of the pandemic. Hospitals, nursing homes, and assisted living facilities also differ in isolation, testing, and exposure protocols. Social media has also made it easier to create and spread COVID-19 misinformation, often competing with sources with proper public health education. As the COVID-19 pandemic was unexpected and new, information is always evolving and causing guidelines to change frequently, which adds a challenge to keeping the most updated information. This creates confusion and fear in health care providers, administration staff, volunteers, families, and patients.

One of the major goals of palliative medicine and hospice health care professionals is to provide the most vulnerable and terminally ill patients with premium care and a dignified death despite the circumstances associated with the pandemic. It is, therefore, essential to better understand the needs of these personnel to assure optimum care, especially in the context of their receiving accurate information. To better define the challenges and needs of palliative medicine and hospice workers, an electronic survey was conducted to employees and volunteers of a large not-for-profit hospice organization in Florida, an area devastated by COVID-19 with a large elderly population.

## Materials and Methods

### Study design

To assess clinical knowledge, attitudes, and beliefs toward COVID-19 infection in palliative medicine and hospice care, a web-based survey was created. Questions were formulated based on a literature review as well as common practices by hospice health care workers. Primary outcomes included attitudes, beliefs, and knowledge. Relevant sociodemographic data were obtained, including age, gender, role at hospice organization, as well as self-reported high risk to develop complications related to COVID-19 infection, level of comfort treating patients with COVID-19, perceived adequacy of personal protective equipment (PPE), sources of information, positive and negative beliefs regarding mitigation guidelines, motivations for behavior toward risk-reduction guidelines, and general concerns regarding the pandemic. Based on the Centers for Disease Control and Prevention (CDC) guidelines at the time of the survey, eight knowledge questions were formulated in a 3-point Likert scale using common clinical scenarios. Secondary outcomes included sociodemographic characteristics, attitudes, and beliefs among health care workers who describe themselves at high risk versus not at high risk of complications related to COVID-19 infection.

### Participants

Hospice employees and volunteers of ages 18 years and older were included. Participants did not receive any direct financial benefit from the proposed research.

### Data collection

A total of 1262 adults were invited to complete the survey from August 17 to September 8, 2020. Invited participants were recruited using an all personnel email without exclusions based on age, gender, and role at the institution. Consent was incorporated into the survey and obtained in English for all participants. Anonymized data for noncommercial research were used. Identifiers were not collected or linked to participants' identities.

### Data analysis

Responders with missing data were excluded. Descriptive statistics were calculated for age, gender, high risk of developing complications, employment status, level of comfort treating patients with COVID-19, PPE, sources of information, beliefs, and knowledge questions.

Statistical analyses were performed using the JMP program Version 15.0.0 Pro (SAS Institute, Cary, NC). A comparison of categorical variables was done using a chi-square test. Results were considered significant if *p*-values were found to be <0.05.

### Ethical considerations

Approval was obtained from the Hospice Quality Assurance and Performance Improvement Department. Institutional review board (IRB) exemption from the University of Miami was obtained. Strict confidentially of data was maintained.

## Results

**FIG. 1.**
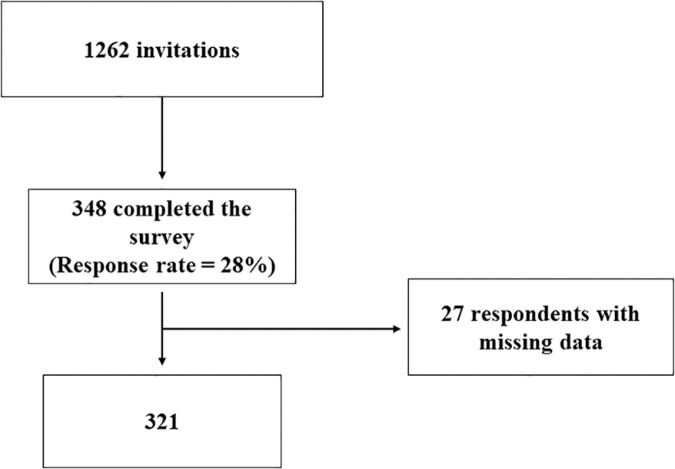
Flowchart of patients recruited for the study.

A total of 348 respondents completed the survey; of these, we analyzed 321 since we excluded the respondents with any missing data (Fig. 1).

The majority of the respondents were females. Out of 321 people, 127 considered themselves at high risk to develop complications related to COVID-19 infection (Table 1).

**Table 1.** Palliative Medicine and Hospice Care Health Care Workers' Sociodemographic Characteristics, Attitudes, and Beliefs

Characteristics, attitudes, and beliefs	n = 321 (%)
Age	
<50	146 (45.48)
>50	175 (54.52)
Gender	
Female	272 (84.74)
Male	47 (14.64)
Nonbinary	1 (0.31)
Prefer not to answer	1 (0.31)
Role at the hospice organization	
Nurse	134 (41.75)
Administration staff	95 (29.60)
CNA	21 (6.54)
Physician	21 (6.54)
Social worker	21 (6.54)
Chaplain	10 (3.12)
Other	10 (3.12)
Volunteer	5 (1.55)
APRN	4 (1.24)
^a^High risk to develop complications	
Yes	127 (39.56)
No	194 (60.44)
Level of comfort	
Very uncomfortable	40 (12.46)
Not comfortable	87 (27.10)
Neutral	112 (34.90)
Comfortable	62 (19.31)
Very comfortable	20 (6.23)
^b^PPE is adequate	
Yes	249 (77.57)
No	72 (22.43)
Source of information	
News	222 (69.16)
Social media	82 (25.55)
Local government	128 (39.88)
Health authorities	272 (84.73)
Health care professionals	214 (66.67)
Workplace	243 (75.70)
Friends	64 (19.94)
Colleagues	99 (30.84)
Research publications	115 (35.82)
^c^Believe in guidelines	
Yes	287 (89.41)
No	34 (10.59)
Risk-reduction strategies	
Hand washing	301 (93.77)
Avoiding nonessential travels, and social gathering	279 (86.92)
Maintaining at least six feet apart	293 (91.28)
Wearing a face mask	297 (92.52)
Wearing an N-95 with all patients	123 (38.32)
Wearing eye protection	197 (61.37)
Motivation for behavior	
Fear of punishment	12 (3.74)
Slowing the spread	226 (70.41)
Avoid bringing the infection to their loved ones	284 (88.47)
Return to normal life	221 (68.85)
Fear of illness/death	167 (52.03)
Other beliefs	
COVID-19 was likely created in a laboratory	101 (31.46)
COVID-19 is a political strategy	54 (16.82)
The contagion is “fake” or overblown	15 (4.67)
The COVID-19 is simply a new flu	58 (18.07)
We live in a “free” society and they have the right to refuse to wear a mask	13 (4.05)
Do not believe in vaccinations	28 (8.72)
Concern the most about COVID-19	
Health system capacity	129 (40.19)
Illness/death of themselves or loved ones	254 (79.13)
Financial impact	194 (60.44)
Spreading COVID to others	226 (70.41)
Social isolation	92 (28.66)
Depression	67 (20.87)
Burn out at work	124 (38.63)
Missing human connection	127 (39.56)

^a^ People who self-reported at high risk for developing complications related to COVID-19 infection.

^b^ People who believe in mitigation guidelines.

^c^ PPE.

COVID-19, coronavirus disease 2019; PPE, personal protective equipment.

The majority of the respondents were incorrect in seven of the eight clinical scenarios (Table 2).

**Table 2.** Clinical Scenarios Based on Centers for Disease Control and Prevention Guidelines to Explore Knowledge

Clinical scenarios	Agree n = 321 (%)	Disagree n = 321 (%)	Unsure n = 321 (%)
Serologic (blood) testing should be used to establish the presence or absence of SARS-CoV-2 infection or reinfection?	172 (53.58)	45 (14.02)^a^	104 (32.40)
Recovered patients can continue to have SARS-CoV-2 RNA detected in their upper respiratory specimens for up to 12 weeks	171 (53.27)^a^	8 (2.50)	142 (44.23)
Do you consider a patient to be contagious until a follow-up negative COVID-19 PCR test (not a rapid test)?	181 (56.39)	65 (20.25)^a^	75 (23.36)
For persons previously diagnosed with symptomatic COVID-19 who remain asymptomatic after recovery, retesting is recommended within three months after the date of symptom onset for the initial COVID-19 infection to ensure cure.	153 (47.66)	62 (19.32)^a^	106 (33.02)
I only trust results from COVID-19 PCR test,“not rapid” test.	69 (21.49)	109 (33.96)^a^	143 (44.55)
Two negative COVID-19 PCR tests should be standard to discontinue isolation and contact precautions.	172 (53.58)	76 (23.68)^a^	73 (22.74)
For most persons with COVID-19 illness, isolation and precautions can generally be discontinued 10 days after symptom onset and resolution of fever for at least 24 hours, without the use of fever-reducing medications and with improvement of other symptoms.	117 (36.45)^a^	91 (28.35)	113 (35.20)
When a patient who initially tested positive for COVID-19 is retested with a “rapid” test and is found to be negative, “I trust the negative result and isolation and contact precautions should be stopped”	41 (12.77)	154 (47.98)^a^	126 (39.25)

^a^ Correct answers based on CDC guidelines at the time of survey.

CDC, Centers for Disease Control and Prevention.

This study found several statistically significant results among the sociodemographic characteristics, attitudes, and beliefs among health care workers who describe themselves at high risk versus not at high risk of complications related to COVID-19 infection (Table 3).

**Table 3.** Analysis of Sociodemographic Characteristics, Attitudes, and Beliefs among Health Care Workers Who Describe Themselves at High Risk versus Not at High Risk of Complications Related to Coronavirus Disease-19 Infection

	High risk n = 127 (%)	Not at high risk n = 194 (%)	Chi-square test	p
Age >50 years	84 (66.14)	91 (46.91)	11.452	0.0007^§^
Female	109 (85.83)	163 (84.02)	2.438	0.4866
Role at the hospice organization				
Nurse	68 (53.54)	66 (34.02)	17.928	0.0218^§^
Administration staff	26 (20.47)	69 (35.57)		
CNA	11 (8.66)	10 (5.15)		
Physician	6 (4.72)	15 (7.73)		
Social worker	7 (5.51)	14 (7.22)		
Chaplain	3 (2.36)	7 (3.61)		
Other	2 (1.57)	8 (4.12)		
Volunteer	2 (1.57)	3 (1.55)		
APRN	2 (1.57)	2 (1.03)		
Level of comfort				
Very uncomfortable	23 (18.11)	17 (8.76)	8.682	0.0696
Not comfortable	38 (29.92)	49 (25.26)		
Neutral	38 (29.92)	74 (38.14)		
Comfortable	22 (17.32)	40 (20.62)		
Very comfortable	6 (4.72)	14 (7.22)		
PPE is adequate	84 (66.14)	165 (85.05)	15.774	<0.0001^§^
Source of information				
News	88 (69.29)	134 (69.07)	0.002	0.9668
Social media	29 (22.83)	53 (27.32)	0.812	0.3676
Local government	53 (41.73)	75 (38.66)	0.302	0.5825
Health authorities	110 (86.61)	162 (83.51)	0.574	0.4488
Health care professionals	81 (63.78)	133 (68.56)	0.788	0.3746
Workplace	101 (79.53)	142 (73.20)	1.673	0.1959
Friends	27 (21.26)	37 (19.07)	0.230	0.6314
Colleagues	44 (34.65)	55 (28.35)	1.426	0.2324
Research publications	44 (34.65)	71 (36.60)	0.127	0.7213
Belief in guidelines	109 (85.83)	178 (91.75)	2.846	0.0916
Risk-reduction strategies				
Hand washing	119 (93.70)	182 (93.81)	0.002	0.9671
Avoiding nonessential travels, and social gathering	119 (93.70)	160 (82.47)	8.506	0.0035^a^
Maintaining at least six feet apart	119 (93.70)	174 (89.69)	1.550	0.2131
Wearing a face mask	120 (94.49)	177 (91.24)	1.173	0.2788
Wearing an N-95 with all patients	60 (47.24)	63 (32.47)	7.084	0.0078^a^
Wearing eye protection	91 (71.65)	106 (54.64)	9.372	0.0022^a^
Motivation for behavior				
Fear of punishment	7 (5.51)	5 (2.58)	1.837	0.1753
Slowing the spread	94 (74.02)	132 (68.04)	1.315	0.2515
Avoid bringing the infection to their loved ones	116 (91.34)	168 (86.60)	1.691	0.1934
Return to normal life	88 (69.29)	133 (68.56)	0.019	0.8895
Fear of illness/death	92 (72.44)	75 (38.66)	35.093	<0.0001^a^
Other beliefs				
COVID-19 was likely created in a laboratory	44 (34.65)	57 (29.38)	0.986	0.3206
COVID-19 is a political strategy	12 (9.45)	42 (21.65)	8.165	0.0043^a^
The contagion is “fake” or overblown	1 (0.79)	14 (7.22)	7.122	0.0076^a^
The COVID-19 is simply a new flu	15 (11.81)	43 (22.16)	5.558	0.0184^a^
We live in a “free” society and they have the right to refuse to wear a mask	1 (0.79)	12 (6.19)	5.756	0.0164^a^
Do not believe in vaccinations	9 (7.09)	19 (9.79)	0.707	0.4006
Concern the most about COVID-19				
Health system capacity	50 (39.37)	79 (40.72)	0.058	0.8092
Illness/death of themselves or loved ones	112 (88.19)	142 (73.20)	10.447	0.0012^a^
Financial impact	74 (58.27)	120 (61.86)	0.413	0.5203
Spreading COVID to others	88 (69.29)	138 (71.13)	0.125	0.7236
Social isolation	32 (25.20)	60 (30.93)	1.233	0.2668
Depression	27 (21.26)	40 (20.62)	0.019	0.8900
Burn out at work	50 (39.37)	74 (38.14)	0.049	0.8254
Missing human connection	41 (32.28)	86 (44.33)	4.658	0.0309^a^

^a^ *p*-value is statistically significant <0.05.

Out of 72 people who felt that PPE was not adequate to protect themselves, 48 felt uncomfortable or very uncomfortable treating patients with COVID-19 infection (*p* ≤ 0.0001). Out of 82 people who used social media as a source of information, 46 were <50 years of age (*p* = 0.0253). Out of 124 who reported fear of burn out at work, 67 were <50 years of age (*p* = 0.0147). Out of 134 nurses, 62 reported fear of burn out at work, compared with 4 out of 21 physicians (*p* = 0.0192).

There was a significant difference in those whose motivation for their behavior was fear of illness/death in the high risk versus the not at high risk group (*p* ≤ 0.0001). There was also a significant difference between these groups related to some of their other beliefs, including COVID-19 being a political strategy (*p* = 0.0043), the contagion being fake or overblown (*p* = 0.0076), COVID-19 being simply a new flu (*p* = 0.0184), and that they have the right to refuse a mask because they live in a free society (*p* = 0.0164).

## Discussion

The findings in this study demonstrate a lack of understanding and knowledge as well as a major element of uncertainty and fear regarding the repercussions of the COVID-19 pandemic among palliative medicine and hospice health care professionals in a large not-for-profit hospice organization. This, in turn, could interfere with the delivery of premium care at the end of life, where families need peace and closure, not turmoil. Effective communication and coordination of up-to-date knowledge among health care personnel is a critical element for successfully managing the COVID-19 pandemic, especially when dealing with frail hospice patients and their families.

Global support for efforts to reduce disease burden is essential. As the pandemic progresses and mitigation strategies evolve, understanding public attitudes, behaviors, and beliefs is critical to implementation of public health policies.^12^ Overall, 89.41% of respondents supported the risk-reduction strategies, whereas 10.59% did not support these measures at the time of the survey. Doing something to mitigate the situation, such as obtaining the most accurate information, can help reduce anxiety, improve quality of care, and potentially help manage the pandemic.

The ongoing COVID-19 pandemic has brought mental, social, and physical suffering. The population that has the highest rate of mortality are those who are elderly, have multiple comorbidities, or are suffering a terminal illness.^13^ In addition, due to overtaxed health care resources and strict infection precautions, a dignified death may be compromised.^14^ Owing to the highly infectious nature of COVID-19, many patients face the end of life in social isolation with no family at the bedside, deprived of the solace provided by the touch or closeness from loved ones.

The COVID-19 pandemic has caused major disruptions in all aspects of daily life, from public to private personal interactions. In addition, major changes occurred overnight in medicine, including reduction of social and physical interactions and cessation of many in-person medical visits.^15^ In this unpredictable evolution of health care delivery systems, providers are being asked to shift from in-person to virtual visits to reduce the spread of COVID-19.^16^ In this study, 39.56% of respondents expressed concerns regarding missing human connections during the pandemic.

Mental health has been a major focus in discussions of how the pandemic is affecting society. The pandemic, mitigation efforts, and economic impact raise the risk of homelessness, substance abuse, depression, anxiety, and suicide.^15^ The prevalence of anxiety and depression in patients with COVID-19 infection was higher in those with pre-existing conditions. Studies from China, Italy, Turkey, Spain, and Iran reported higher-than-pooled prevalence between health care workers and the general public.^17^ Female health care workers and nurses had showed higher rates of affective symptoms than male and medical staff, respectively.^18^ Other risk factors included social isolation.^17^ This study found a significant percentage of hospice employees and volunteers who expressed concern about burn out at work (38.63%), social isolation (28.66%), and depression (20.87%). Historically, a “one-size-fits-all” approach has been the mainstay of psychological support for health care workers exposed to disasters, limited to the immediate postresponse phase.^19^ The concept of resilience has gained significant attention in recent times.^20^ Other protective factors included having sufficient medical resources, up-to-date and accurate information, and taking precautionary measures.^17^ This study aims to promote mental well-being and resilience, develop psychological interventions targeting high-risk personnel, and improve staff knowledge related to COVID-19 by training health care workers with up-to-date information. The health and wellness committee is offering free virtual stretching, yoga, and fitness classes to encourage exercise during the COVID-19 pandemic. The hospice organization is offering free COVID-19 online courses with accurate information.

Availability and use of appropriate PPE arguably represent one of the most significant challenges that has faced health care systems during the COVID-19 pandemic. Issuing clear PPE guidance and ensuring adequate supply of appropriate PPE for health care workers have been an enormous task placed on the government and health care organizations. Criticism has been rampant in the mainstream media, focusing on shortages of PPE for frontline health care workers, with some staff stating that their lives are at risk due to PPE failings.^21^ Many nursing homes are diverting resources to stop the spread of the coronavirus, but most have inadequate resources for a sufficient supply of PPE for residents and staff.^22^ This study found statistically significant difference in the perception of adequacy of available PPE in between palliative medicine and hospice employees and volunteers who considered themselves at high risk of complications compared with those who did not consider themselves at high risk. It was observed that most of participants who felt that the provided PPE was insufficient to protect themselves felt uncomfortable or very uncomfortable treating patients with COVID-19 infection. Evaluating staff awareness of PPE guidance, educating health care professionals on how to safely use it, and dedicating special attention to high-risk personnel could improve staff safety, as well as minimize fear and anxiety. This, in turn, could translate to better quality of care for vulnerable patients.

Some of our palliative medicine and hospice personnel provided care for patients at long-term care (LTC) facilities. The COVID-19 pandemic has disproportionately affected residents and staff at LTC facilities in the United States, with high case fatality rates.^23,24^ This emphasizes the need for continued contact restrictions, increased testing of residents and staff, and strict infection control policies, including increased access to PPE for staff.^25^ Given that this relatively small percentage of the population comprises a disproportionally large percentage of morbidity and mortality from COVID-19, providing appropriate education to, and understanding the perception of, the health care workers is vital to optimizing care of these patients.

This study had several limitations. First, attitudes and beliefs were self-reported; therefore, responses might be subject to social desirability bias. Second, knowledge questions were based on the CDC guidelines at the time of the survey; thus, the findings might have limited generalizability.

## Conclusions

Throughout most of this COVID-19 pandemic, the majority of discussion and attention has been focused on vaccine development, critical care treatments, and rehabilitation. Very limited attention has been directed to the needs and perceptions of the health care workers who provide the care at the end of life. As the COVID-19 crisis continues, one priority should be developing interventions to assure appropriate education to this critical element of the health care system. At the same time, emphasis should be given to monitoring for depression, anxiety, and burn out. Improving staff knowledge and information related to COVID-19 would enhance staff safety, improve patient care, and relieve anxiety.

## Abbreviations Used

CDC: Centers for Disease Control and Prevention
COVID-19: coronavirus disease 2019
LTC: long-term care
PPE: personal protective equipment
